# Fast spiking interneurons autonomously generate fast gamma oscillations in the medial entorhinal cortex with excitation strength tuning ING–PING transitions

**DOI:** 10.1101/2025.09.05.674527

**Published:** 2025-09-05

**Authors:** Brandon Williams, Ananth Vedururu Srinivas, Roman Baravalle, Fernando R. Fernandez, Carmen C. Canavier, John. A. White

**Affiliations:** 1 Department of Biomedical Engineering, Center for Systems Neuroscience, Neurophotonics Center, Boston University, Boston, MA, 02215, USA; 2 Department of Cell Biology and Anatomy, Louisiana State University Health Sciences Center, New Orleans, LA, 70112, USA

## Abstract

Gamma oscillations (40–140 Hz) play a fundamental role in neural coordination, facilitating communication and cognitive functions in the medial entorhinal cortex (mEC). While previous studies suggest that pyramidal-interneuron network gamma (PING) and interneuron network gamma (ING) mechanisms contribute to these oscillations, the precise role of inhibitory circuits remains unclear. Using optogenetic stimulation and whole-cell electrophysiology in acute mouse brain slices, we examined synaptic input and spike timing in neurons across layer II/III mEC. We found that fast-spiking interneurons exhibited robust gamma-frequency firing, while excitatory neurons engaged in gamma cycle skipping. Stellate and pyramidal cells received minimal recurrent excitation, whereas fast-spiking interneurons received strong excitatory input. Both excitatory neurons and fast-spiking interneurons received gamma frequency inhibition, emphasizing the role of recurrent inhibition in gamma rhythm generation. Notably, gamma activity persisted after AMPA/kainate receptor blockade, indicating that interneurons can sustain gamma oscillations independently through an ING mechanism. Selective activation of PV+ interneurons confirmed their ability to sustain fast gamma inhibition autonomously. To further assess the interplay of excitation and inhibition, we developed computational network models constrained by our experimental data. Simulations revealed that weak excitatory input to interneurons supports fast ING-dominated rhythms (~100–140 Hz), while strengthening excitatory drive induces a transition to slower PING-dominated oscillations (60–80 Hz). These findings highlight the dominant role of inhibitory circuits in sustaining gamma rhythms, demonstrate how excitation strength tunes the oscillatory regime, and refine models of entorhinal gamma oscillations critical for spatial memory processing.

## Introduction

Cortical network oscillations play a fundamental role in coordinating neural activity (Buzsáki and Draguhn, 2004; Fries, 2009), facilitating communication between brain regions (Colgin, 2013), and supporting cognitive functions such as spatial navigation and memory (Buzsáki and Moser, 2013). In the medial entorhinal cortex (mEC), grid cells—neurons that exhibit spatially periodic firing—are strongly modulated by theta (4–12 Hz) and gamma (40–140 Hz) oscillations. Theta rhythms regulate the timing of grid cell firing (Fyhn et al., 2004; Hafting et al., 2005, 2008) and enable hippocampal-entorhinal interactions (Buzsáki and Moser, 2013). Gamma rhythms have been implicated in sensory processing, attention, and working memory (Colgin et al., 2009). However, the cellular and synaptic mechanisms underlying theta-nested gamma oscillations in mEC, particularly the contributions of excitatory and inhibitory circuits, remain incompletely understood.

Gamma oscillations are hypothesized to arise from local circuit interactions involving both excitatory principal neurons and inhibitory interneurons. Two primary mechanisms have been proposed: pyramidal-interneuron network gamma (PING), which relies on excitatory-inhibitory interactions (Traub et al., 1996; Börgers and Kopell, 2003; Wang, 2010; Buzsáki and Wang, 2012), and interneuron network gamma (ING), which emerges from mutual inhibition among fast-spiking interneurons (Whittington et al., 2000; Bartos et al., 2007; Buzsáki and Wang, 2012). These models are not mutually exclusive and may operate in parallel or under different network conditions. Disentangling their respective contributions requires experiments that can isolate the roles of excitatory and inhibitory populations while monitoring gamma rhythms.

Pharmacological evidence supports both PING and ING mechanisms. Blockade of AMPA/kainate receptors significantly reduces gamma power throughout the hippocampal–entorhinal system (Pastoll et al., 2013; Butler et al., 2018), consistent with a role for fast excitatory input in gamma generation (i.e., PING). However, in the mEC, gamma oscillations often persist following AMPA/kainate receptor blockade, suggesting that inhibitory synchronization (ING) may also be sufficient. This raises the possibility that the mEC employs both PING and ING mechanisms, although this remains unresolved.

**Extended Data Figure 1-1:**
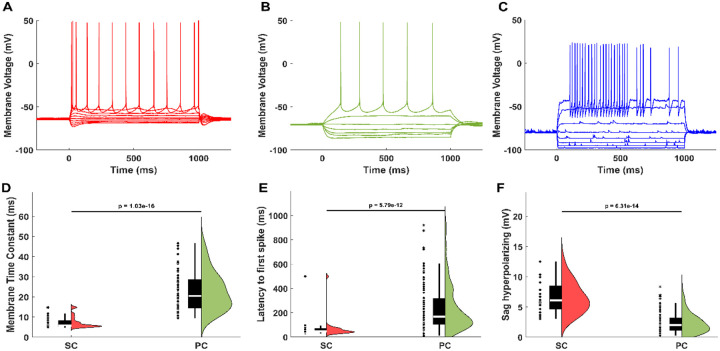
Classification of major electrophysiological cell types in mEC. A) Example voltage response to current steps in stellate cell. B) Example voltage response to current steps in pyramidal cell. C) Example voltage response to current steps in fast-spiking interneuron. D) Membrane time constants of stellate and pyramidal cells. Stellate cells have shorter time constants. E) Latency to first spike of stellate and pyramidal cells. Stellate cells fire sooner than pyramidal cells. F) Hyperpolarizing sag potential in stellate and pyramidal cells. Stellate cells have larger hyperpolarizing sag potentials.

Principal neurons in layer II/III of mEC consists of stellate and pyramidal cells (Alonso and Klink, 1993; Canto and Witter, 2012) with distinct electrophysiological profiles (Extended Data Fig. 1-1). Grid cells, predominantly found among stellate and pyramidal neurons (Sargolini et al., 2006; Domnisoru et al., 2013), have been proposed to rely on recurrent excitatory connectivity (Fuhs and Touretzky, 2006; McNaughton et al., 2006). However, anatomical studies indicate that excitatory connectivity is sparse (Dhillon and Jones, 2000; Couey et al., 2013; Fuchs et al., 2016), particularly among stellate cells, raising the possibility that inhibitory circuits play a central role in shaping grid cell dynamics.

Parvalbumin-positive (PV+) fast-spiking interneurons, which constitute approximately 50% of the inhibitory population in mEC (Wouterlood et al., 1995; Miettinen et al., 1996), also have a distinct electrophysiological profile (Extended Data Fig. 1-1) and are critical for gamma rhythm generation (Sohal et al., 2009). These interneurons provide fast, perisomatic inhibition to grid cells (Buetfering et al., 2014; Miao et al., 2017), yet the extent of their recurrent connectivity remains debated. One study reported strong chemical and electrical coupling (Fernandez et al., 2022), whereas another found only gap junctions (Huang et al., 2024). Clarifying this connectivity is essential to understanding whether PV+ interneurons can autonomously generate gamma oscillations.

In this study, we use optogenetic stimulation, whole-cell recordings, pharmacological manipulations, and computational modeling to test whether fast-spiking interneurons in the mEC can generate gamma oscillations independently. We compare the timing and strength of inhibitory input across cell types, assess interspike intervals and phase-locking to theta-frequency drive, and evaluate the persistence of gamma oscillations following blockade of AMPA/kainate receptors. In parallel, we develop network simulations constrained by our experimental data to examine how excitatory-inhibitory balance regulates gamma rhythms. These combined approaches aim to determine whether inhibitory circuits in the mEC are sufficient to sustain gamma rhythms (ING) or whether excitatory-inhibitory interactions are required, as predicted by PING models, and how excitation strength tunes the transition between these mechanisms.

## Materials and methods

All procedures were approved by the Institutional Animal Care and Use Committee of Boston University and conformed to NIH guidelines. Adult (2–6 months old) male and female mice were used in roughly equal numbers. A transgenic mouse line constitutively expressed ChR2 in both excitatory and inhibitory cells under the Thy1 promoter (Thy1-ChR2-EYFP, JAX strain #007612). A separate transgenic mouse line expressed Cre recombinase under the PV promoter (PV-Cre, JAX strain #017320). Parvalbumin is expressed almost exclusively in fast-firing inhibitory interneurons in MEC. The transgenic PV-Cre mouse line was cross bred with transgenic LoxP-ChR2-EYFP mice (JAX, strain #024109) to express ChR2 in PV+ fast-spiking interneurons (PV-ChR2) after Cre-Lox recombination.

### Acute slice preparation

All measurements were obtained from 400-micron thick horizontal mouse brain slices. Bilateral slices from the dorsomedial mEC (~3.2–4.3 mm from the dorsal surface of the brain) were used. Mouse brains were extracted after isofluorane overdose and decapitation. During slicing, the brain was submerged in sucrose-substituted artificial cerebrospinal fluid (ACSF) solution (in mM: sucrose 185, KCl 2.5, NaH_2_PO_4_ 1.25, MgCl_2_ 10, NaHCO_3_ 25, glucose 12.5, and CaCl_2_ 0.5) at 4°C that was continuously perfused with 95% oxygen / 5% carbon dioxide (carboxy) gas and sliced with a vibratome (VT1200, Leica Microsystems). Then, brain slices are moved to a separate chamber containing standard ACSF (in mM: NaCl 125, NaHCO_3_ 25, D-glucose 25, KCl 2, CaCl_2_ 2, NaH_2_PO_4_ 1.25, and MgCl_2_ 1) perfused with carboxy gas and incubated at 37°C for 30 minutes. After incubation, the brain slices are allowed to recover for 15 minutes at room temperature (~20°C) where they remained until being used for whole-cell electrophysiology.

### Whole-cell electrophysiology

For electrophysiology experiments, brain slices were continuously perfused with ACSF at a temperature of 35–37°C. The ACSF was bubbled with 95%/5% carboxy gas throughout the experiments. Borosilicate glass pipettes were pulled (Sutter Instrument P-97) and filled with intracellular fluid solution (in mM: K-gluconate 120, KCl 20, HEPES 10, diTrisPhCr 7, Na_2_ATP 4, MgCl_2_ 2, TrisGTP 0.3, and EGTA 0.2, and buffered to pH 7.3 with KOH) and pipettes with resistances of 4–8 MΩ were used for electrophysiology recordings. Pipette capacitance was neutralized in the on-cell configuration, and series resistance was compensated to at least 50%. Using voltage clamp, excitatory and inhibitory post-synaptic currents were recorded at −70 and 0 mV, respectively. Liquid junction potentials were not corrected. Electrophysiology data was amplified using Axon Instruments MultiClamp 700B and sampled at 30 kHz using Axon Instruments DigiData 1440A. Custom protocols were designed using pClamp 7.0 software to control data collection and optogenetic stimulation. Pipettes and cells were visualized with diffuse interference contrast (DIC). A 470 nm LED (Thorlabs, M470L4) delivered widefield optogenetic stimulation through a 40x objective lens with a typical intensity of ~2 mW/mm^2^, although up to 12 mW/mm^2^ was used to probe connectivity. During pharmacological experiments, an AMPA antagonist (5 μM DNQX) was allowed to wash in for at least 10 minutes before repeating electrophysiological recordings.

### Cell type classification

The major electrophysiological cell types in the mEC were statistically separated by their electrophysiological properties (Extended Data Fig. 1-1). Current steps were injected from −200 pA to 525 pA at 25 pA intervals to characterize the subthreshold and firing properties of each cell. Putative stellate and pyramidal cells in layer II/III mEC were classified based on membrane sag potential and membrane time constant as used previously (Fernandez et al., 2022). Fast-spiking interneurons were easily identified by their discontinuous membrane current – spike rate relationship, high threshold firing, burst firing, fast membrane time constant, and short spike half width.

### Data analysis and statistics

All data analysis was performed with custom written algorithms in MATLAB. Action potential peaks were detected and registered to the phase of the stimulation period. Spike phase histograms were computed with 30 equally spaced bins over the stimulation period (width = π/15 radians) and were normalized by the number of theta cycles (40 cycles for each cell). Interspike frequency histograms were computed with 10 Hz bins and the bin counts were normalized by the number of theta cycles (40 cycles for each cell).

For current recordings, raw data was forward and reversed filtered from 50–200 Hz with 4th order butterworth filters. To analyze the frequency and amplitude of gamma oscillations, the continuous wavelet transform was computed in MATLAB. The analytic morlet wavelet (ω_0_ = 6 rad/sec) was used to calculate the scalogram for each theta cycle with 32 scales per octave. The network activity during the first stimulation period varied greatly compared to subsequent cycles and was removed from analysis. The magnitude of the scalogram was averaged over the next 40 theta cycles to determine the peak gamma frequency, phase and power of the membrane currents for each cell. Any theta cycles with large current artifacts or spikes (> 3000 pA) were removed from analysis. Low average peak gamma power recordings (< 20 pA^2^ for Thy1 and < 10 pA^2^ for PV experiments), scalograms peaks with large bandwidths (> 100 Hz for Thy1 and > 110 Hz for PV experiments), and scalograms with peak powers less than five multiples above the average (SNR < 5) were removed for accurate gamma frequency comparisons.

Non-parametric methods were used for statistical testing to control for non-normality and differences in sample sizes unless otherwise noted. For paired recordings obtained before and after the application of DNQX, the two-sided Wilcoxon signed-rank test was used to compare the change in gamma power or frequency. The Kruskal-Wallis test was used for comparisons between independent cell types. A significant effect was followed by a post-hoc Dunn’s test with Bonferroni correction for multiple comparisons. Significance was determined at p < 0.05.

### Computational methods

All simulations were carried out using the NetPyNE (Dura-Bernal et al., 2019) simulator based on Python. The network model and associated programs to run simulations can be found on https://github.com/AnanthVS23/mEC_Network_Simulations. The network consists of 500 single compartment model neurons of two populations: 100 inhibitory PV+ interneurons and 400 excitatory stellate cells. The optogenetic theta drive was simulated by a sinusoidal 8 Hz conductance waveform biased to have only positive values with a peak of 7 nS for the PV cells and 3 nS for the stellate cells and a reversal potential of 0 mV. Five readout neurons from each population were voltage-clamped at 0 mV to measure inhibitory currents during theta drive. The frequency and power of oscillations in the inhibitory currents were analyzed using the analytic morlet wavelet in the neuro digital signal processing package of Python to obtain the scalogram.

#### PV+ interneuron model:

The model consisting of 100 PV+ interneurons is based on our previous work (Via et al., 2022), calibrated using data based on the passive and intrinsic properties of these neurons (Fernandez et al., 2022). A small amount of noise was added to the theta drive to the PV+ cells to prevent synchronization by the theta drive alone in the hyperpolarizing case.

#### Stellate cell model:

The model for the stellate cells (SCs) is based on the original Hodgkin-Huxley conductance-based model (Hodgkin and Huxley, 1952) modified to better capture the resonance properties of the stellate cells in the mEC (Alonso and Klink, 1993). Heterogeneity is introduced by jittering the conductance values from a Gaussian distribution to obtain 400 stellate cell models.

#### Synapses:

Inhibitory synapses onto the SCs were calibrated with a rise time constant of 0.4 ms and decay time constant of 6 ms, with E_GABA_ = −65 mV (Sauer et al., 2012). The SCs in this model are not connected with each other, based on the experimental data (Couey et al., 2013). Excitatory synapses to the PV+ interneurons use E_AMPA_ = 0 mV and an exponential synapse with a decay time constant of 1 ms.

#### Connectivity:

The probability of I–I connections was as in (Via et al., 2022). The probability of E–I was 40% and I-E connectivity was 30%. The weights of the I–E connections were also randomized using a lognormal distribution as in (Fernandez et al., 2022). To ensure that the results were reproducible, and not an artefact of the connectivity parameters for a single simulation, the simulations were run with 10 different seeds to randomize the connectivity. The results across the simulations were found to be consistent (Fig. 6).

## Results

A transgenic mouse line was used to express ChR2 primarily in stellate cells and fast-spiking interneurons in layer II/III mEC. However, pyramidal neurons have also been shown to express ChR2 in mEC with the Thy1 promoter (Proskurina and Zaitsev, 2021). Acute brain slices were prepared from roughly equal numbers of adult male and female Thy1-ChR2-EYFP mice. Whole-cell patch clamp recordings were obtained from stellate, pyramidal and fast-spiking interneurons in layer II/III mEC during 8 Hz theta-frequency sinusoidal optogenetic stimulation, to simulate network drive observed in spatial navigation.

### Firing characteristics of stellate, pyramidal and fast-spiking interneurons during theta-frequency Thy1+ stimulation

**Figure 1:**
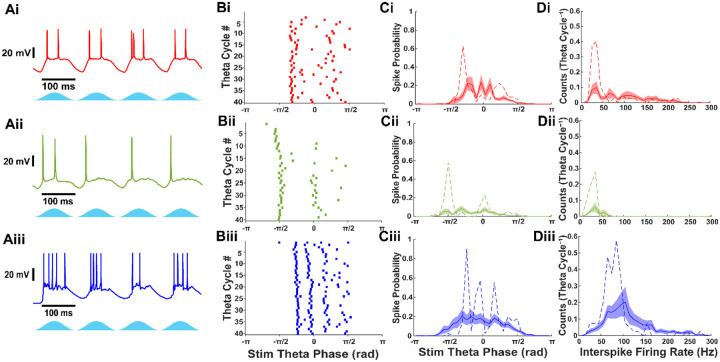
Stellate, pyramidal, and fast-spiking interneurons exhibit different firing patterns during theta frequency optogenetic stimulation of Thy1+ neurons. A) Current clamp recordings of (i) stellate, (ii) pyramidal, and (iii) fast-spiking interneurons during theta frequency optogenetic stimulation of Thy1+ neurons. B) Raster plots during 40 theta cycles of optogenetic stimulation from the cells shown in A. C) Average histogram of theta stimulation spike phase for stellate, pyramidal and fast-spiking interneurons. Examples from A and B are shown as dashed line. Shaded region indicates S.E.M. D) Average histogram of interspike firing rate distribution for stellate, pyramidal, and fast-spiking interneurons. Examples from A and B are shown as dashed line. Shaded region indicates S.E.M.

Voltage recordings were analyzed to characterize the firing responses of different neuron types during optogenetic stimulation of Thy1+ neurons (Fig. 1A). All cell types exhibited firing activity during stimulation. However, pyramidal neurons demonstrated a lower probability of activation (77.1%, n = 27/35) compared to stellate (94.4%, n = 17/18) and fast-spiking interneurons (88.9%, n = 16/18). The firing rates varied among the neuron types. Stellate and pyramidal neurons fired an average of 1.54 ± 0.28 and 0.67 ± 0.12 spikes per theta cycle, respectively, while fast-spiking interneurons exhibited a higher firing rate of 2.40 ± 0.58 spikes per theta cycle. The spike phase distribution for stellate cells showed peaks corresponding to fast gamma frequencies (Fig. 1C), and individual stellate cells occasionally fired at ~100 Hz (Fig. 1D). However, most stellate neurons displayed interspike intervals that were slower multiples of the network frequency, indicative of gamma cycle skipping. Pyramidal neurons, in contrast, were unable to fire at fast gamma frequencies and instead skipped one or two gamma cycles between action potentials. Fast-spiking interneurons exhibited a broader spike-phase distribution, with some firing multiple times per putative gamma cycle, while the majority fired at fast gamma frequencies. These results highlight distinct cell-type-specific firing dynamics in response to theta-frequency stimulation, with fast-spiking interneurons most consistently engaging in fast gamma activity, stellate cells exhibiting intermittent participation and cycle skipping, and pyramidal neurons showing limited involvement, suggesting differential contributions to gamma rhythm generation in layer II/III mEC. We note that the reduced activity of pyramidal neurons could be due to limited expression of ChR2 under the Thy1 promoter (Pastoll et al., 2013).

### Fast-spiking interneurons receive strong gamma frequency excitatory input during Thy1+ stimulation

Previous studies have suggested that stellate cells have minimal recurrent connectivity in layer II mEC (Dhillon and Jones, 2000; Couey et al., 2013; Pastoll et al., 2013), while pyramidal neurons exhibit slightly higher connectivity (Fuchs et al., 2016). Notably, fast-spiking interneurons receive gamma-frequency excitatory currents during theta-frequency optogenetic stimulation of Thy1+ neurons (Pastoll et al., 2013). However, these measurements included a mix of excitatory and inhibitory currents, as voltage was clamped at −50 mV. To isolate fast excitatory inputs, we performed voltage-clamp recordings at −70 mV, thereby minimizing inhibitory and excluding NMDA-mediated currents while capturing AMPA-mediated and ChR2 currents.

**Figure 2:**
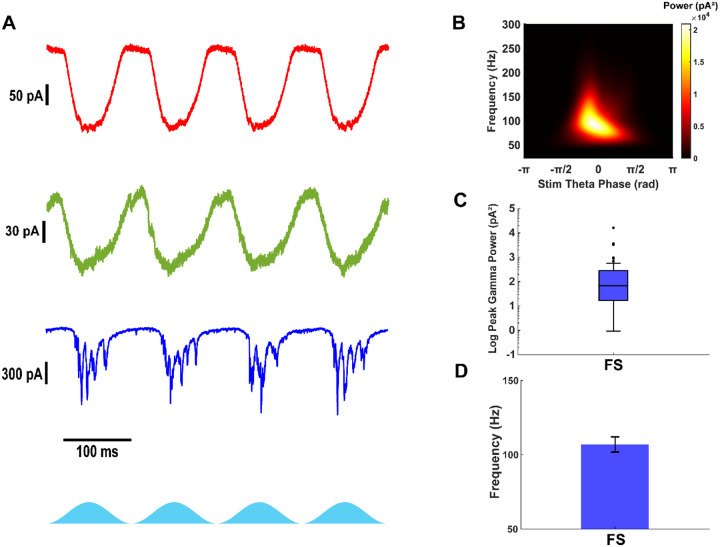
Only fast-spiking interneurons receive fast and strong gamma frequency excitatory currents. A) Example excitatory currents from stellate (red), pyramidal (green) and fast-spiking interneurons (blue) during theta-frequency optogenetic stimulation of Thy1+ neurons. B) Average scalogram of gamma frequency excitatory currents in fasts-piking interneurons during 40 theta cycles of optogenetic stimulation. C) Peak gamma power from scalograms of excitatory currents in fast-spiking interneurons. D) Gamma frequency at the peak gamma power from scalograms of excitatory currents in fast-spiking interneurons.

Our recordings revealed that fast-spiking interneurons received AMPA-mediated excitatory currents at fast gamma frequencies in addition to theta-frequency ChR2 currents (Fig. 2A, 2B). Whereas all stellate cells exhibited ChR2-induced excitatory currents, they did not display fast outward currents during Thy1+ stimulation, indicating negligible recurrent excitation. Due to strong inhibitory input and imperfect space clamp, inward currents were partially observed even at −70 mV. Most pyramidal cells expressed ChR2 currents and small excitatory post-synaptic currents (EPSCs), but these synaptic events lacked robust oscillations. In contrast, fast-spiking interneurons received strong gamma-frequency excitation during optogenetic stimulation (Peak gamma power: 68 pA^2^ (17 – 281), n = 38, Fig. 2C; Gamma frequency: 106.9 ± 5.1 Hz, n = 19, Fig. 2D), suggesting that they are uniquely recruited by local excitatory cells and well-positioned to provide gamma frequency inhibition, as demonstrated by their firing rates in Fig. 1.

### Excitatory cells receive stronger inhibition than fast-spiking interneurons during Thy1+ stimulation

Fast-spiking interneurons are key generators of gamma frequency activity, but how different excitatory and inhibitory cell types receive and integrate inhibitory input remains unclear. Given the distinct connectivity patterns and intrinsic properties of stellate, pyramidal, and fast-spiking interneurons, we sought to determine how inhibitory input varies across these cell types during optogenetically driven network activity.

**Figure 3:**
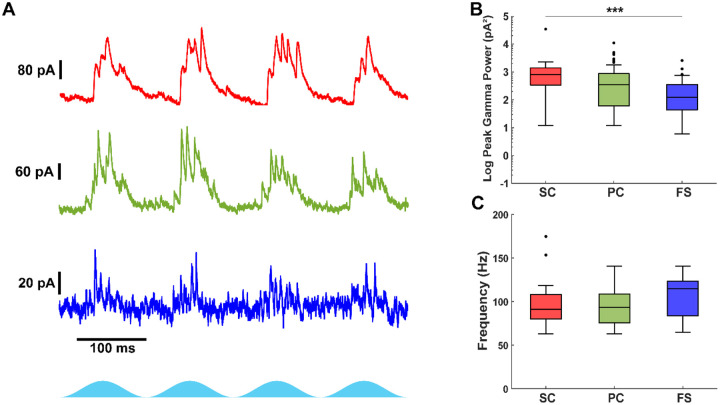
Excitatory neurons receive stronger gamma-frequency inhibitory input than fast-spiking interneurons. A) Example inhibitory current recordings in stellate (red), pyramidal (green) and fast-spiking interneurons (blue) during optogenetic stimulation of Thy1+ neurons. B) Comparison of the peak gamma power from the average scalogram of each cell. Gamma frequency inhibition is weaker in fast-spiking interneurons. C) Comparison of the peak gamma frequency from the average scalogram of each cell. Filled circles indicate outliers.

To compare inhibitory input across neuron types, we voltage-clamped neurons at 0 mV to minimize excitatory synaptic and ChR2 currents. All cell types received fast gamma frequency inhibitory currents (Fig. 3A). Cell type had a significant effect on the peak inhibitory gamma power (χ^2^(2,125) = 14.22, p = 8.2e−4, Kruskal–Wallis test). Stellate cells received stronger inhibitory gamma currents than fast-spiking interneurons (Stellate: 823 pA^2^ (339 – 1399), n = 20; Fast-spiking: 124 pA^2^ (44 – 352), n = 34; p = 5.8e−4; Fig. 3B), but their inhibition was comparable to that of pyramidal neurons (Pyramidal: 346 pA^2^ (60 – 870), n = 74; p = 0.079). The inhibitory current power was nearly significantly different between pyramidal neurons and fast-spiking interneurons (p = 0.053). In general, stellate and pyramidal cells exhibited greater summation of inhibitory currents per theta period compared to fast-spiking interneurons. However, cell type did not have a significant effect on the frequency of inhibitory currents (χ^2^(2,95) = 5.06, p = 0.079, Kruskal–Wallis test). Therefore, fast-spiking interneurons received inhibition at a similar frequency compared stellate (Fast-spiking: 114.4 Hz (83.7 – 123.4), n = 16; Stellate: 91.1 Hz (80.0 – 107.8), n = 19; Fig. 3C) and pyramidal neurons (Pyramidal: 93.1 Hz (75.4 – 108.4), n = 63). These findings confirm that both excitatory cells and fast-spiking interneurons receive gamma frequency inhibition, although excitatory neurons integrate stronger and more prolonged inhibition compared to fast-spiking interneurons.

### Fast-spiking interneurons generate gamma frequency inhibition without AMPA-mediated excitation

Models of grid cells and theta-nested gamma oscillations in the medial entorhinal cortex often assume a role for recurrent excitation. However, previous connectivity studies have shown that stellate cells exhibit minimal recurrent excitation (Dhillon and Jones, 2000; Couey et al., 2013; Pastoll et al., 2013), while pyramidal cells display a higher probability of recurrent connectivity (Fuchs et al., 2016). As a result, alternative grid cell models propose that principal neurons are connected exclusively through recurrent inhibition. It is crucial to quantify the extent of AMPA-mediated excitation in layer II/III of the mEC to refine grid cell models.

**Figure 4:**
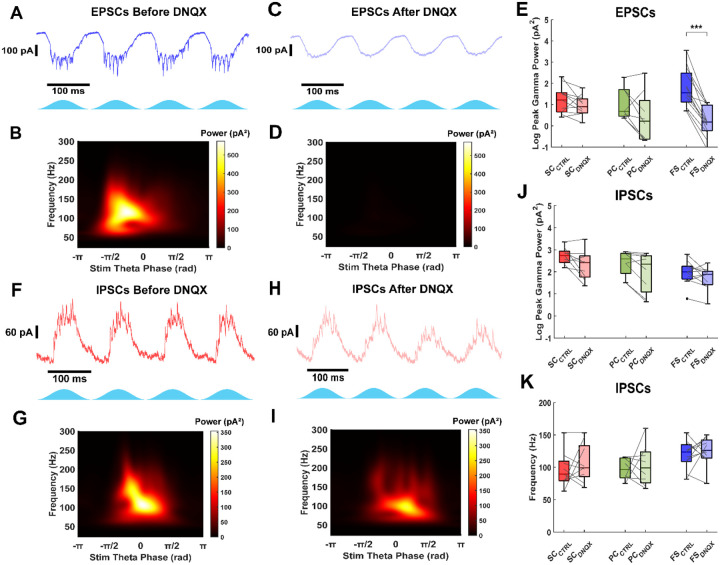
Thy1+ fast-spiking interneurons provide fast and strong inhibition in the absence of fast excitatory synaptic input. A) Example excitatory current trace in fast-spiking interneuron during Thy1 stimulation. B) Average scalogram of 40 theta cycles of excitatory currents from trace in A. Data was filtered from 50 to 200 Hz. C) Excitatory currents recorded from the same fast-spiking interneuron as A after blocking AMPA mediated synaptic input with 5 μM DNQX. D) Average scalogram of 40 theta cycles of excitatory currents from trace in C. Data was filtered from 50 to 200 Hz. E) Comparison of excitatory current peak gamma power for all cell types before and after DNQX. Gamma frequency currents are abolished in fast-spiking interneurons. F) Example inhibitory current trace in stellate cell before blocking AMPA receptors with DNQX. G) Average scalogram from 40 theta cycles of inhibitory currents from trace in F. H) Inhibitory currents recorded from the same stellate cell in F after blocking AMPA receptors with DNQX. I) Average scalogram from 40 theta cycles of inhibitory currents from trace in H. J) Comparison of inhibitory current peak gamma power for all cell types before and after DNQX. The inhibitory current power doesn’t significantly change with synaptic excitation blocked. K) Comparison of inhibitory current gamma frequency for all cell types before and after DNQX. The frequency of inhibition doesn’t significantly change with synaptic excitation blocked. Gray lines indicate paired data points.

To isolate AMPA-mediated excitation, we recorded excitatory currents before and after applying the AMPA receptor antagonist DNQX (5 μM). Blocking AMPA receptors abolished strong gamma-frequency excitatory currents in fast-spiking interneurons (Fig. 4A–E). Peak gamma power in excitatory currents significantly decreased in fast-spiking interneurons (Median log difference: −1.48 (−1.85 – −1.17), n = 13, p = 2.4e−4, Wilcoxon signed-rank test), while it remained unchanged in pyramidal neurons (Median log difference: −0.98 (−1.19 – −0.01), n = 8, p = 0.11, Wilcoxon signed-rank test) and stellate cells (Median log difference: −0.35 (−0.50 – 0.03), n = 11, p = 0.10, Wilcoxon signed-rank test). The cell type had a significant effect on the change in peak gamma power (χ^2^(2,29) = 15.69, p = 3.9e−4, Kruskal–Wallis test). The reduction in peak gamma power for fast-spiking interneurons was significantly different compared to stellate (p = 4.0e−4) and pyramidal (p = 0.033) cells, but no significant difference was found between stellate and pyramidal cells (p = 1). These findings indicate that fast-spiking interneurons, but not stellate or pyramidal cells, receive AMPA excitatory inputs, consistent with anatomical studies.

Pastoll et al. (2013) previously demonstrated that gamma frequency inhibitory currents in stellate cells significantly decreased after blocking both AMPA and NMDA excitatory currents but remained unchanged when only NMDA currents were blocked. This suggests that AMPA excitation plays a crucial role in sustaining strong gamma frequency activity in the mEC. To determine whether AMPA-mediated excitation is necessary for gamma frequency inhibition, we applied DNQX (5 μM) and recorded synaptic inhibitory currents before and after drug application.

During baseline theta-frequency optogenetic stimulation, all cell types received gamma-frequency inhibitory currents (e.g., Fig. 4F, G), as in our previous findings (Fig. 3). After blocking AMPA-mediated excitation, gamma-frequency inhibitory oscillations persisted across all cell types (Fig. 4H–K). Peak gamma power did not change significantly in stellate cells (Median log difference: −0.15 (−1.01 – 0.02), n = 9, p = 0.10, Wilcoxon signed-rank test), pyramidal neurons (Median log difference: −0.41 (−0.81 – −0.07), n = 8, p = 0.055, Wilcoxon signed-rank test), or fast-spiking interneurons (Median log difference: −0.23 (−0.51 – 0.09), n = 11, p = 0.21, Wilcoxon signed-rank test; Fig. 4J). Additionally, the median frequency of these inhibitory oscillations remained unchanged across all groups (Median difference: Stellate: 0 Hz (−17.58 – 30.98), n = 9, p = 0.74; Pyramidal: −4.53 Hz (−21.99 – 28.21), n = 8, p = 0.95; Fast-spiking: 11.42 Hz (−21.82 – 21.89), n = 11, p = 0.64, Wilcoxon signed-rank test; Fig. 4K). Interestingly, these results suggest that fast-spiking interneurons can generate gamma-frequency inhibition independently of AMPA-mediated excitatory input. There was, however, considerable variability between experiments (see Fig. 4K). In some cases, the frequency changed very little, implying an ING mechanism (e.g., Fig. 4F–I). In others, the frequency increased substantially or decreased slightly after blocking AMPA receptors. The mEC apparently wires itself up to produce fast gamma oscillations when presented with theta drive, but this can be achieved in multiple ways. Neurons have homeostatic mechanisms that allow them to produce their characteristic electrical activity resulting in a large variability (2–5 fold) in the specific values for each conductance in different neurons of the same type (Goaillard and Marder, 2021). This phenomenon is called degeneracy (Golowasch et al., 2002) and also applies to circuits. We use computational modeling below to account for some of this variability.

### PV+ fast-spiking interneurons generate fast gamma frequency inhibition in the mEC

Because our findings showed that fast gamma-frequency inhibition persisted even in the absence of AMPA-mediated synaptic input, we sought to determine whether driving PV+ fast-spiking interneurons alone could generate fast gamma-frequency inhibition and whether their activity differed from broader network-driven inhibition. To investigate this, we crossbred PV-Cre and LoxP-ChR2-EYFP transgenic mouse lines to express ChR2 in PV+ interneurons (PV-ChR2). Whole-cell recordings were used to capture voltage activity and synaptic currents during theta frequency optogenetic stimulation of PV+ interneurons.

**Figure 5:**
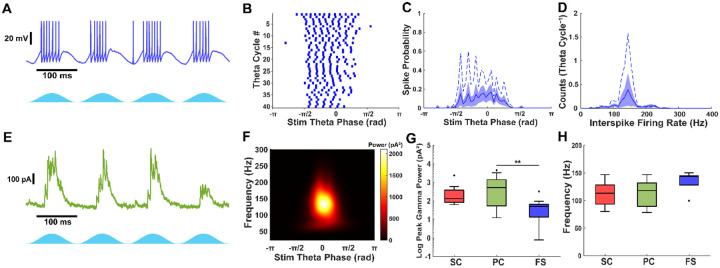
PV+ inhibitory current oscillations are weaker and faster than Thy1. A) Fast-spiking interneuron voltage trace during optogenetic stimulation. B) Raster plot of 40 theta cycles from voltage trace in A. C) Stimulation spike-phase histogram of fast-spiking interneurons. Solid line and shaded region show mean ± S.E.M. Dashed line represents example shown in A, B. D) Interspike firing rate histogram of fast-spiking interneurons. Solid line and shaded region show mean ± S.E.M. Dashed line represents example shown in A, B. E) Pyramidal cell inhibitory current trace during theta-frequency PV+ stimulation. F) Average scalogram from 40 theta cycles of inhibitory currents from trace in F. G) PV+ inhibitory current peak gamma power in all cell types. Inhibition is stronger in excitatory cells. PV+ Inhibition power in stellate and fast-spiking interneurons is weaker than Thy1 stimulated inhibition. H) PV+ inhibitory current gamma frequency. PV+ inhibition (ING) is faster than Thy1+ (PING). Outliers are indicated by filled circles.

We found that PV+ fast-spiking interneurons could fire at fast gamma frequencies during theta-frequency optogenetic stimulation (Fig. 5A, B). Some fast-spiking interneurons fired during optogenetic stimulation (50%, n = 2/4) with an average of 4.34 ± 1.83 spikes per theta cycle for active interneurons. The distribution of theta phase spiking was broadly tuned over the center half of the stimulation period (Fig. 5C), as with Thy1+ stimulation. The interspike firing rate of PV+ fast spiking interneurons peaked at approximately 150 Hz (Fig. 5D), exceeding the peak firing frequency observed during Thy1+ stimulation.

**Extended Data Figure 5-1:**
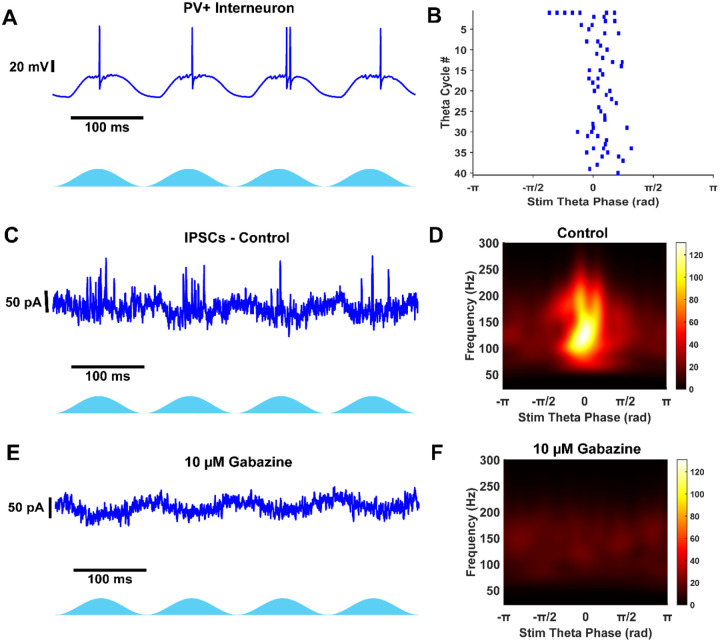
PV+ interneuron receives fast GABAergic theta-nested gamma inhibition. A) Voltage recording in PV+ interneuron during network PV+ optogenetic stimulation. B) Raster plot from 40 theta stimulation periods in same cell as A. C) Voltage clamp at 0 mV records theta-nested IPSCs during PV+ stimulation. D) Average Scalogram from 40 theta stimulation periods of data from example in C. E) Voltage clamp at 0 mV observes no IPSCs after blocking GABA_A_ receptors with 10 μM Gabazine. This data verifies theta-nested gamma in C is GABAergic inhibition. F) Average scalogram from 40 theta stimulation periods after blocking GABA_A_ channels. Gamma frequency activity is abolished.

All cell types received fast gamma frequency PV+ inhibition during optogenetic stimulation (Fig. 5E, F, H), including PV+ interneurons (Extended Data Fig. 5-1). Cell type had a significant effect on the peak gamma power of PV+ inhibitory currents (χ^2^(2,41) = 10.92, p = 0.0043, Kruskal–Wallis test). Pyramidal cells received the strongest gamma frequency inhibition (Pyramidal: 531 pA^2^ (55 – 1429), n = 23), significantly greater than fast-spiking interneurons (Fast-spiking: 51 pA^2^ (14 – 66), n = 12; p = 0.0039; Fig. 5G), but not stellate cells (Stellate: 135 pA^2^ (84 – 385), n = 9; p = 1). However, the power of gamma frequency PV+ inhibition was not statistically different between stellate cells and fast-spiking interneurons (p = 0.055), in contrast to what was observed during Thy1+ stimulation. The median peak inhibitory gamma power in stellate cells and fast-spiking interneurons, but not pyramidal cells, was greater during Thy1+ stimulation (Stellate: p = 0.018; Pyramidal: p = 0.76; Fast-spiking: p = 0.011; Mann-Whitney U test), indicating that PV+ interneurons generate weaker inhibitory oscillations compared to network drive of Thy1+ excitatory and inhibitory interneurons.

All cell types received inhibition at similar gamma frequencies during PV+ stimulation (Stellate: 113.2 Hz (93.4 – 128.3), n = 9; Pyramidal: 118.2 Hz (89.2 – 131.7), n = 22; Fast-spiking: 143.6 Hz (128.0 – 145.2), n = 5; χ^2^(2,33) = 5.14, p = 0.077, Kruskal–Wallis test). The median frequency of inhibitory currents was faster during PV+ stimulation in pyramidal cells and fast-spiking interneurons, but not stellate cells compared to Thy1+ stimulation (Stellate: p = 0.11; Pyramidal: p = 1.1e−4; Fast-spiking: p = 0.014; Mann-Whitney U test), indicating that PV+ interneurons synchronize at a slightly faster rate independently.

In one example, we show that optogenetic simulation of PV+ interneurons inhibits the firing of a PV+ cell (Extended Data Fig. 5-1A, B). Furthermore, this PV+ interneuron receives similar high gamma frequency PV+ inhibition compared to excitatory cells (~130 Hz; Extended Data Fig. 5-1C, D). We verified that these currents were GABAergic by washing in 10 μM Gabazine. The blockade of GABA_A_ receptors clearly abolishes gamma frequency currents during optogenetic stimulation (Extended Data Fig. 5-1E, F). The presence of local PV+ synaptic connectivity contrasts with a previous study that found no synaptic connections between PV+ interneurons in layer II mEC (Huang et al., 2024), but supports another that found both synaptic and gap junction connectivity (Fernandez et al., 2022).

These findings demonstrate that PV+ fast-spiking interneurons can independently generate gamma-frequency inhibition and play a central role in maintaining gamma oscillations in the mEC. Furthermore, the faster, weaker inhibition generated during PV+ stimulation suggests a specialized role for PV+ interneurons in modulating local network dynamics to support spatial and memory-related computations.

### Computational modeling of Thy1+ network stimulation in layer II/III mEC

There are two prevailing models (Bartos et al., 2007) for the generation of gamma oscillations, interneuronal network gamma (ING) and pyramidal interneuronal network gamma (PING). In ING, the oscillations are generated by interactions between the interneurons (Wang and Buzsáki, 1996). There is a stochastic ING mechanism in which individual interneurons are subthreshold and fluctuation-driven (Brunel and Hansel, 2006). However, we will not address this mechanism here since the interneurons are suprathreshold due to the theta drive. In PING, the interneurons are generally assumed to be quiescent (Börgers and Kopell, 2003) but are driven to fire by a volley of firing in the excitatory neuron population when that population recovers from the previous wave of inhibition. Although there are two types of excitatory cells in the mEC, stellate and pyramidal cells, for the purpose of our computational model, we will simply call the excitatory cells (‘E cells’), and the inhibitory PV+ interneurons (‘I cells’). We added 400 excitatory (E) stellate cells to our previously calibrated heterogeneous network of 100 fast-spiking interneurons (Via et al., 2022) and did not incorporate a separate model of the pyramidal cell population. The stellate cells were not connected to each other (Couey et al., 2013).

**Extended Data Figure 6-1:**
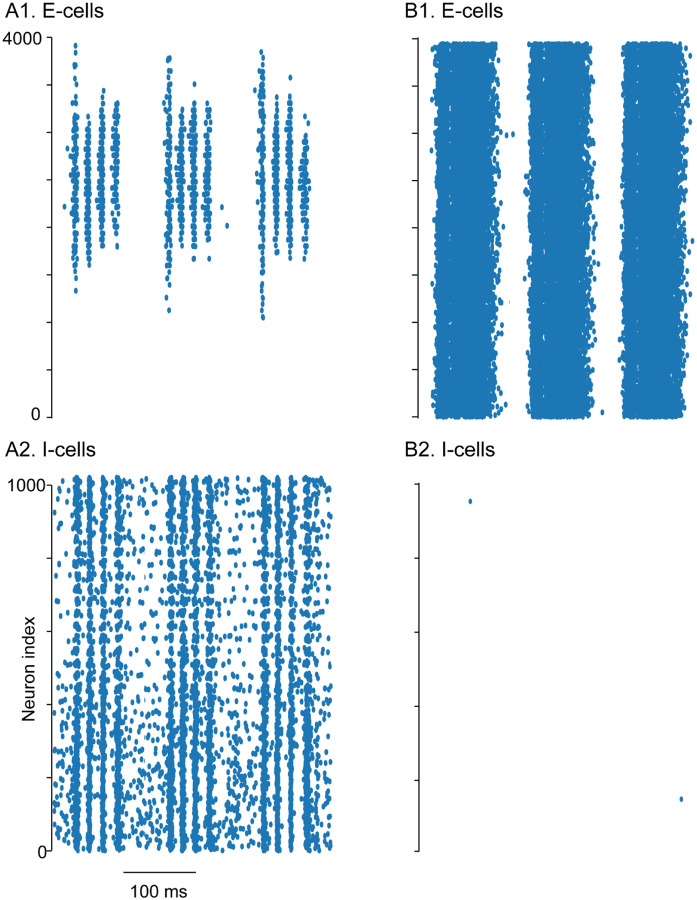
Simulations of theta-nested gamma oscillations using the grid cell model from Pastoll et al. (2013) with and without AMPA-mediated connections. A) Raster plots for (1) excitatory cells and (2) inhibitory cells with the default parameters. B) Raster plots with g_ampa_total set to zero to turn off excitatory synapses. Original model: https://modeldb.science/150031?tab=2&file=GridCellModel/grid_cell_model and a revised simulation_fig_model.py file available at https://github.com/ccanav/pastoll_et_al_2013.

Our computational simulations focused on the relative roles of excitation and inhibition in generating theta-nested gamma in mEC. Previously, optogenetically-driven theta-nested gamma oscillations (~100 Hz) in mEC slices from Thy1-ChR2 mice were abolished after blocking AMPA synaptic currents (Pastoll et al., 2013), in contrast to our results showing a minimal reduction. Interestingly, Fig. S3D of the same study (Pastoll et al., 2013) clearly shows that blocking excitation did not abolish interneuronal firing, contrary to classic models of PING (Börgers and Kopell, 2003) that require the interneurons to be silent in the absence of synaptic excitation, and as assumed by their model of gamma generation (Extended Data Fig. 6-1). Therefore, in our model, the interneurons receive sufficient simulated optogenetic theta drive to fire in the absence of synaptic excitation.

During Thy1+ optogenetic stimulation, fast-spiking interneurons receive both theta frequency ChR2 currents and gamma frequency AMPA-mediated synaptic currents when voltage-clamped at −70 mV. To estimate the physiological range of E to I connections, we measured the peak-to-peak amplitude of the gamma frequency excitatory currents from 5 voltage-clamped fast-spiking interneurons. Two examples of these excitatory current traces can be found in Fig. 2A (bottom) and Fig. 4A. We then divided these currents by the holding potential (−70 mV) to obtain a range of physiological E to I maximal synaptic conductances (5–10 nS). To replicate the experimental data in this study, we used hyperpolarizing GABA_A_ synapses (E_GABA_=−75mV) between interneurons, similar to a previous model (Pastoll et al., 2013).

### E–I conductance strength governs gamma frequency and mechanism

**Figure 6:**
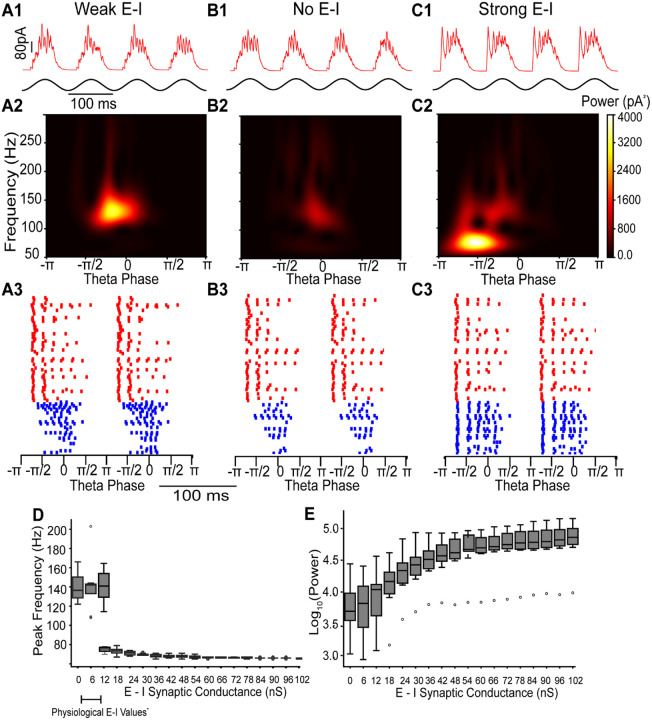
Simulation of Excitatory-Inhibitory network: transition from ING dominated to PING dominated. A) E–I network with weak E to I conductance (3.6 nS). B) E–I network without E to I conductance to simulate AMPA receptor blockade. C) E–I network with strong E to I conductance (18 nS). Row 1: Inhibitory synaptic currents (red) from a representative E cell during simulated optogenetic theta drive (black). Row 2: Average scalogram of inhibitory synaptic currents from Row 1. Row 3: Example raster plots from E cell (red) and I cell (blue). D, E) Simulations were run on ten networks with a different seed for wiring the random connectivity of the network to generate box and whisker plots in which open circles indicate outliers. D) The peak frequency discontinuously transitions from a fast ING dominant rhythm to a slower PING dominant rhythm as the E to I synaptic conductance is increased. E) The power at the peak frequency increases and plateaus as the E to I synaptic conductance is increased.

We first examined the effect of weak E to I connections (3.6 nS) on network synchrony (Fig. 6A). Fig. 6A1 shows simulated inhibitory synaptic currents received by a readout E cell voltage-clamped at 0 mV while the E–I network was driven by simulated 8 Hz optogenetic input. The inhibitory currents were comparable in amplitude to the representative experimental trace in Fig. 4F. The corresponding scalogram (Fig. 6A2) showed peak gamma power at ~125 Hz, closely matching the frequency observed in Fig. 4G. The spike raster (Fig. 6A3) revealed that I cells were recruited following initial E cell activation. However, the I cells did not remain phase-locked to E cell firing and instead synchronized primarily among themselves to generate fast oscillations. To further test the mechanism, we removed the E–I conductance to simulate application of DNQX (Fig. 6B2). This manipulation did not alter the peak frequency, though it slightly reduced the power, consistent with an ING-driven rhythm. In contrast, we found that a strong E-I conductance (18 nS) generated slower gamma frequency oscillations, reflecting a PING dominant rhythm (Fig. 6C).

**Figure 7:**
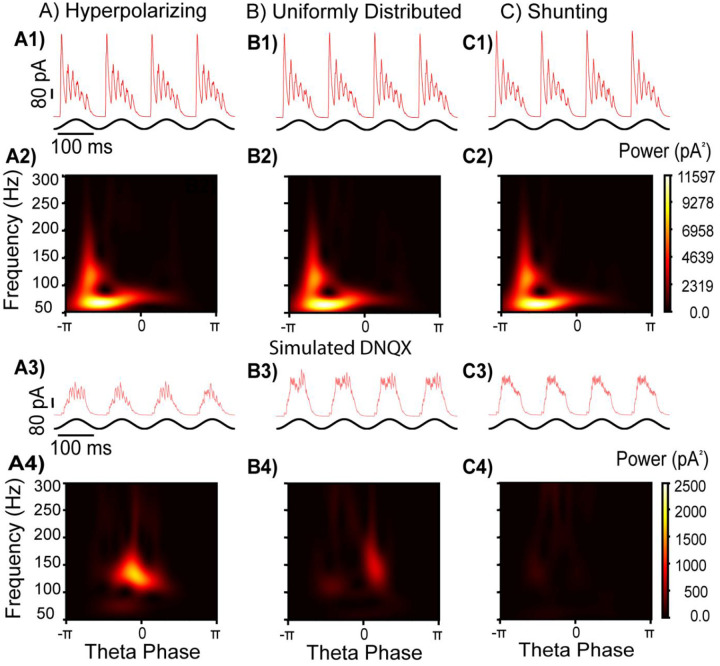
Effect of GABA_A_ reversal potential during simulated AMPA block of extremely strong E to I connections. A) Hyperpolarizing I to I connectivity (E_GABA_=−75 mV). B) I to I connectivity with E_GABA_ uniformly distributed between −75 mV and −55 mV. C) Shunting I to I connectivity (E_GABA_=−55 mV). Row 1: Example inhibitory synaptic currents in a voltage-clamped E cell with strong network E to I connectivity. Row 2: Average scalogram of inhibitory synaptic currents from Row 1. Row 3: Example inhibitory synaptic currents in a voltage-clamped E cell without network E to I connectivity. Row 4: Average scalogram of inhibitory synaptic currents in Row 3. Note that the scale for power is different in simulated AMPA block compared to control.

We next varied the strength of the average total E–I conductance in the network. The connectivity between I to I, E to I and I to E cells was random with fixed probabilities (see Methods), and ten networks with different random seeds were simulated. We found that for conductance values below 12 nS, including the physiological range, a high frequency (~140 Hz peak) frequency oscillation is generated with power independent of E–I connection strength (Fig. 6D). However, at exactly 12 nS, depending upon the connectivity pattern, either a fast ING or a slower PING dominated oscillation is manifested. This slower rhythm arose from recruitment of a population spike in interneurons after the initial volley of excitatory cell activity (Fig. 6C3). The resulting additional inhibition, which manifests as bursts in interneurons that previously fired only once, slowed the oscillation frequency. Nevertheless, a residual higher-frequency component persisted (Fig. 6C3), and its timing advanced within the theta cycle as E–I strength increased (Fig. 7). This conductance-dependent switch in dominant frequency may provide a mechanism for transitions between fast and slow gamma, both of which are observed in the entorhinal cortex (EC): fast gamma in medial EC (Colgin et al., 2009) and slow gamma in lateral EC (Fernández-Ruiz et al., 2021). The same mechanism also explains much of the variability seen in Fig. 4K, aside from decreases in frequency that may reflect experimental variability, or the influence of interneuron subtypes not included in the model (e.g., disinhibition of PV+ cells).

### Influence of GABA_A_ reversal potentials and E–I balance on gamma oscillations

In our models, we utilized connectivity parameters between I cells previously established in our lab (Fernandez et al., 2022; Via et al., 2022). However, another study (Huang et al., 2024), from the same lab that originally reported optogenetically driven theta-nested gamma in the mEC (Pastoll et al., 2013), found gap junctional connectivity but failed to find chemical synaptic connectivity between PV+ interneurons in layer II mEC, in contrast to previous work in our lab (Fernandez et al., 2022). Besides I–I connectivity, the reversal potential of the GABA_A_ synapses mediated by chloride ion concentrations, has a large impact on the synchronization tendencies on interneuronal networks (Wang and Buzsáki, 1996; Via et al., 2022; Baravalle and Canavier, 2024). In dentate gyrus PV+ interneurons, these synapses were shown to be shunting (Vida et al., 2006). However, in CA1 interneurons the GABA_A_ reversal potential varies considerably, ranging from −75 to −55 mV at the soma, with dendrites exhibiting even more hyperpolarized values (Otsu et al., 2020).

To reconcile the differences between our results and those of Pastoll et al. (2013), we next examined how varying GABA_A_ reversal potentials shape network activity under conditions of strong E–I connectivity. First, we simulated hyperpolarizing inhibition between I cells (Fig. 7A), similar to Fig. 6A but with E–I conductance increased to 120 nS to represent the slower, PING-dominated regime. Under these conditions, blocking excitatory synaptic transmission significantly reduced the power of gamma oscillations while increasing their frequency (100–150 Hz). Distributing the reversal potentials between −75 and −55 mV as in (Otsu et al., 2020) produced similar results (Fig. 7B), with an even greater reduction in gamma power. However, shunting inhibition between I cells completely abolished theta-nested gamma activity (Fig. 7C), offering a potential explanation for the divergent results reported by Pastoll et al. (2013).

## Discussion

Our findings provide novel insights into the mechanisms underlying gamma oscillations in the medial entorhinal cortex (mEC), emphasizing the role of fast-spiking interneurons in generating and maintaining these rhythms. By leveraging optogenetic stimulation, whole-cell recordings, pharmacological manipulations, and computational modeling, we systematically examined how excitatory and inhibitory neuronal populations contribute to gamma oscillation dynamics.

Experimentally, we found minimal recurrent excitation in pyramidal and stellate cells, consistent with previous studies (Couey et al., 2013; Fuchs et al., 2016). Gamma-frequency inhibition persisted after blocking AMPA receptors, supporting an interneuron network gamma (ING) mechanism, and selective activation of PV+ interneurons confirmed their ability to autonomously generate fast gamma rhythms. Complementing these results, our computational modeling demonstrated that weak excitatory input sustains fast ING, while stronger excitation promotes a transition to slower pyramidal-interneuron network gamma (PING). This dual mechanism provides a potential basis for the coexistence and dynamic switching of fast and slow gamma oscillations observed in the entorhinal cortex.

### Distinct firings patterns of stellate, pyramidal, and fast-spiking interneurons during Thy1+ stimulation

Our results reveal clear distinctions in the firing properties of stellate, pyramidal, and fast-spiking interneurons during theta-nested gamma oscillations. Fast-spiking interneurons exhibited the highest firing rates and were more likely to fire at fast gamma frequencies (~100 Hz), whereas stellate and pyramidal neurons displayed lower firing rates and engaged in gamma cycle skipping. These findings align with previous studies indicating that fast-spiking interneurons are key pacemakers of gamma oscillations by providing rhythmic inhibition to excitatory cells (Pastoll et al., 2013). This suggests that stellate and pyramidal cell populations can alternate to drive gamma-frequency excitation onto fast-spiking interneurons.

### Recurrent inhibition plays a dominant role in generating gamma oscillations

Voltage-clamp recordings demonstrated that fast-spiking interneurons receive robust AMPA-mediated excitatory currents at gamma frequencies, unlike stellate and pyramidal neurons, which exhibited minimal gamma-frequency excitation. This supports preferential recruitment of fast-spiking interneurons, consistent with dense excitatory input onto PV+ cells (Beed et al., 2013; Couey et al., 2013). The stronger excitatory drive to interneurons suggests that models of grid cells and theta-nested gamma oscillations in mEC should rely solely on excitatory connectivity to interneurons rather than recurrent excitation. Our computational modeling supports this conclusion, as networks with no excitatory-excitatory connectivity still produced robust fast gamma oscillations.

One of the most striking findings in our study is that gamma-frequency inhibitory currents persisted across all neuronal populations even after AMPA receptor blockade with DNQX. This suggests that interneurons in the mEC can generate gamma-frequency inhibition independently of fast excitatory input, a property that distinguishes mEC from other cortical areas where gamma oscillations typically depend on excitatory drive (Buzsáki and Wang, 2012). This finding aligns with the ING model of gamma generation, where mutual inhibition among fast-spiking interneurons can sustain rhythmic activity without excitatory input. Consistently, our simulations showed that interneuron networks alone can sustain fast gamma rhythms, underscoring the role of PV+ interneurons as core gamma generators.

### PV+ fast-spiking interneurons as autonomous gamma generators

Optogenetic activation of PV+ interneurons confirmed their capacity to generate fast gamma-frequency inhibition independently. PV+ interneurons fired at higher frequencies than during Thy1+ stimulation, and all cell types received fast gamma-frequency inhibition during PV+ stimulation. The inhibitory currents were significantly stronger in pyramidal cells compared to fast-spiking interneurons, indicating that PV+ interneurons provide robust inhibition to excitatory neurons. However, these gamma oscillations were faster and weaker compared to Thy1+ stimulation, suggesting that other interneuron types could be recruited through feedback inhibition. Additionally, we demonstrate that fast-spiking interneurons receive fast synaptic inhibition from PV+ interneurons (Fig. 5, Extended Data Fig. 5-1), aligning with a previous study that found high levels of both synaptic and electrical coupling between PV+ interneurons (Fernandez et al., 2022), although other reports have found only gap junctions (Huang et al., 2024). These results support the notion that PV+ interneurons play a central role in coordinating gamma oscillations and maintaining rhythmic inhibitory drive in the mEC (Buetfering et al., 2014). Since theta oscillations in the hippocampal formation are generally thought to be mediated by theta-rhythmic GABAergic inhibition of PV+ interneurons (Buzsáki, 2002), there is likely a baseline level of excitation of these neurons *in vivo* during theta-nested gamma oscillations for the theta modulation to be effective.

### Reconciling divergent results: a novel PING mechanism and shunting inhibition

One possible explanation for the differences between our findings and Pastoll et al. (2013) is that the chemical synaptic connections between inhibitory cells were weaker or nonexistent in their study as described in (Huang et al., 2024) from the same lab. However, the distances between most of the PV cells in Huang et al. (2024) exceed 200 μm, where we would expect minimal synaptic connectivity (Fernandez et al., 2022). A more plausible explanation could be differences in the reversal potential of GABA_A_ synapses, which significantly impacts the synchronization of interneuronal networks (Wang and Buzsáki, 1996; Via et al., 2022; Baravalle and Canavier, 2024). The extracellular solutions used in Pastoll et al. (2013) and our study had similar concentrations of chloride and bicarbonate ions. However, the intracellular concentration of these two ions in the unmonitored neurons which generate the gamma oscillations in slice preparations are unknown. Higher concentrations of intracellular chloride or bicarbonate (Farrant and Kaila, 2007) could have rendered GABA_A_ synapses shunting, accounting for the stronger effect of AMPA block.

Our simulations of a shunting network (Fig. 7C) suggest a novel form of PING in which interneurons fire asynchronously without synaptic excitation but are forced to synchronize when excitatory input is active. The theoretical basis for this form of PING (Chandrasekaran et al., 2011) was recently extended to account for synaptic delays (Vedururu Srinivas and Canavier, 2024). The principle is that the E–I network can be reduced to a 2D discrete map based on slopes of the phase resetting curves (PRC). In a shunting network that cannot synchronize itself, perturbations grow because they are multiplied by a scaling factor greater than one. The slope of the PRC is stabilized by the input from the excitatory cell and enforces synchrony. This novel PING dominated mechanism does not require inhibitory cells to be quiescent in the absence of synaptic excitation (Extended Data Fig. 6-1), as for classic PING (Börgers and Kopell, 2003).

### Implications for grid cell function and spatial computation

Our findings have important implications for understanding how grid cells process spatial information. The persistence of gamma oscillations despite blocking fast excitatory input suggests that inhibitory circuits are crucial for maintaining grid cell dynamics. Given that grid cells rely on precise timing of excitatory and inhibitory inputs, our data suggest that PV+ interneurons may regulate spatial coding by imposing temporal constraints on excitatory neuron firing. Many computational models of grid cell activity and theta-gamma coupling have focused on reciprocal excitatory-inhibitory circuits (Couey et al., 2013; Pastoll et al., 2013). Our results support the addition of inhibitory-inhibitory connectivity to these models, which has been shown to enhance grid stability and increase gamma frequency (Solanka et al., 2015; Shipston-Sharman et al., 2016).

The two major models of grid cell activity are single bump and multi-bump models (Shipston-Sharman et al., 2016). Single bump models require synaptic profiles with surround connectivity strongest at about half the sheet width, whereas multi-bump models rely on shorter-distance connections. According to Figure 74 in (Paxinos and Franklin, 2001), the lateral mEC is about 0.8 by 2 mm at its largest extent. In rats, grid modules extended across mediolateral band for distances greater than 1 mm (Stensola et al., 2012). Based on our previous results (Fernandez et al., 2022), chemical synapses between PV+ interneurons, between SST+ interneurons, and chemical synapses from PV+ and SST+ onto excitatory neurons are minimal at distances greater than 100–200 μm. Moreover, our results show that stellate and pyramidal cells receive minimal gamma frequency excitation (Fig. 2), arguing against recurrent synaptic excitation. Therefore, the only possible source of long-range connectivity to support single bump models is from excitatory to inhibitory cells, which has not yet been extensively characterized.

### Conclusion and future directions

In summary, our study provides compelling evidence that fast-spiking interneurons are essential for gamma oscillation generation in the mEC. While AMPA-mediated excitation plays a role in driving interneuron activity, gamma-frequency inhibition persists independently of excitatory input, suggesting a dominant role for inhibitory circuits. PV+ interneurons serve as autonomous generators of gamma rhythms, orchestrating network-wide inhibitory dynamics. Complementing these findings, computational modeling revealed that the strength of excitatory input tunes the balance between ING- and PING-dominated regimes, offering a mechanistic explanation for the coexistence and potential switching between fast and slow gamma oscillations. These findings refine existing models of mEC function and emphasize the importance of inhibitory networks in spatial computation and memory processing.

Further studies should explore how gamma oscillations in the mEC interact with other brain regions involved in spatial navigation, such as the hippocampus. Additionally, computational modeling approaches could help elucidate the precise contribution of inhibitory networks to spatial coding. Investigating gamma oscillation disruptions in neurodegenerative diseases like Alzheimer’s may also provide valuable insights into the role of inhibitory dysfunction in cognitive decline.

## Extended Data

## Significance Statement

Gamma oscillations in the medial entorhinal cortex (mEC) are essential for spatial navigation and memory, yet the mechanisms underlying their generation remain unresolved. Combining optogenetics, whole-cell electrophysiology, and computational modeling, we show that fast-spiking interneurons autonomously sustain gamma rhythms via interneuron network gamma (ING). Blocking excitatory input did not abolish gamma-frequency inhibition, and selective activation of PV+ interneurons confirmed their capacity to generate gamma independently. Modeling revealed that excitatory strength tunes the oscillatory regime, with weak excitation favoring fast ING and stronger excitation inducing slower pyramidal-interneuron network gamma (PING). These findings refine entorhinal gamma models and suggest a dynamic mechanism for switching between fast and slow gamma critical for spatial computation.
